# On human nanoscale synaptome: Morphology modeling and storage estimation

**DOI:** 10.1371/journal.pone.0310156

**Published:** 2024-09-25

**Authors:** Wieslaw L. Nowinski

**Affiliations:** Sano Centre for Computational Personalised Medicine, Kraków, Poland; Nanjing University, CHINA

## Abstract

One of the key challenges in neuroscience is to generate the human nanoscale connectome which requires comprehensive knowledge of synaptome forming the neural microcircuits. The synaptic architecture determines limits of individual mental capacity and provides the framework for understanding neurologic disorders. Here, I address morphology modeling and storage estimation for the human synaptome at the nanoscale. A synapse is defined as a pair of pairs [(presynaptic_neuron),(presynaptic_axonal_terminal);(postsynaptic_neuron),(postsynaptic_dendritic_terminal)]. Center coordinates, radius, and identifier characterize a dendritic or axonal terminal. A synapse comprises topology with the paired neuron and terminal identifiers, location with terminal coordinates, and geometry with terminal radii. The storage required for the synaptome depends on the number of synapses and storage necessary for a single synapse determined by a synaptic model. I introduce three synaptic models: topologic with topology, point with topology and location, and geometric with topology, location, and geometry. To accommodate for a wide range of variations in the numbers of neurons and synapses reported in the literature, four cases of neurons (30;86;100;138 billion) and three cases of synapses per neuron (1,000;10,000;30,000) are considered with three full and simplified (to reduce storage) synaptic models resulting in total 72 cases of storage estimation. The full(simplified) synaptic model of the entire human brain requires from 0.21(0.14) petabytes (PB) to 28.98(18.63) PB for the topologic model, from 0.57(0.32) PB to 78.66(43.47) PB for the point model, and from 0.69(0.38) PB to 95.22(51.75) PB for the geometric model. The full(simplified) synaptic model of the cortex needs from 86.80(55.80) TB to 2.60(1.67) PB for the topologic model, from 235.60(130.02) TB to 7.07(3.91) PB for the point model, and from 285.20(155.00) TB to 8.56(4.65) PB for the geometric model. The topologic model is sufficient to compute the connectome’s topology, but it is still too big to be stored on today’s top supercomputers related to neuroscience. *Frontier*, the world’s most powerful supercomputer for 86 billion neurons can handle the nanoscale synaptome in the range of 1,000–10,000 synapses per neuron. To my best knowledge, this is the first big data work attempting to provide storage estimation for the human nanoscale synaptome.

## 1. Introduction

The human brain is an enormously complex, multi-scale, and multi-level structure operating in space and time as well as generating molecular, cellular, and neuronal phenomena [1]. Understanding its structure, function, and dysfunction is a huge scientific challenge, a critical social need, and a great market opportunity. While attempting to solve emerging problems, neuroscience progresses tremendously producing big data [2] whose processing requires high-performance computing [3]. The challenges emerge in the acquisition, processing, modeling, interpretation, storage, and use of neurodata; here we address modeling and storage.

The knowledge of connectome is essential for understanding how neural microcircuits encode information and how the brain works in health and disease [4]. The full nanoscale connectomes have only been completed for nematode *Caenorhabditis elegans* (*C*.*elegans*) [5], larva *Ciona intestinalis* [6], and *Drosophila* [7]. These animals have very small brains; namely, *Ciona intestinalis* 177 neurons and about 6,600 connections, *C*.*elegans* 302 neurons (a constant number) and about 7,000 connections, and *Drosophila* about 100–130 thousand neurons and millions of connections. The human connectome has been developed at the macroscale by providing anatomical and functional connectivity [8].

The full human nanoscale connectome, however, has not yet been created. The most critical obstacles in obtaining it are the prohibitively long overall time necessary to acquire data and the computing resources required for storing and processing these big data [2,3,9]. For instance, to image the whole human brain would require an estimated 17 million years [9] by applying the same brain imaging protocol as was employed for *Drosophila* [7]. In addition, it is assessed that the complete reconstruction of 1 mm^3^ of the cortex would require 10,000 man-years [10] and cost above $100 million [4].

Owing to the progress in imaging, the acquisition time constantly decreases. In particular, synchrotron X-ray tomography [11] is a promising imaging modality able to decrease the whole human brain acquisition time at the sub-cellular level to a few years [12] or even less. At present, for instance, by employing phase-contrast synchrotron tomography, whole brain imaging was performed in about 16 hours at a 25 μm spatial resolution with 16-bit voxels [13]. This level of spatial resolution enables imaging the neuronal cell bodies, however, it still is insufficient to reveal the complete neurons with their dendrites and axons as well as the synapses that functionally connect neurons.

The creation of the nanoscale human connectome requires the segmentation of all neurons and the reconstruction of all neuronal microcircuits forming it, each microcircuit considered a sequence of synapses, so consequently comprehensive knowledge of synaptome, i.e., the complete set of all synapses in the brain. The synaptic architecture determines the limits of individual mental capacity and provides the framework for understanding neurologic disorders [14].

The goal of this work is to 1) formulate three (topology-, location-, and geometry-related) morphological models of the human synaptome at the nanoscale along with their simplified versions to reduce storage and 2) estimate storage requirements for these models. To accommodate for a wide range of variations in the numbers of neurons and synapses reported in the literature, four cases of the total number of neurons in the brain (30;86;100; and 138 billion) and three cases of the number of synapses per neuron (1,000;10,000; and 30,000) are considered with three full and the corresponding simplified synaptic models resulting in total 72 cases of storage estimation. To my best knowledge, this is the first big data work providing the storage requirement estimation for the human nanoscale synaptome.

## 2. Material and method

The storage required for the synaptome can be calculated as

singlesynapsestoragexsynapsenumber (1)

The number of synapses can be approximated by half of the product of the average number of neurons by the average number of synapses per neuron (a pair of the connected neurons form a synapse by the presynaptic and postsynaptic neurons, so to avoid duplication in synapse counting half of the product shall be taken)


$$\begin{array}{l} single\;synapse\;storage\;x\;0.5\;average\;number\;of\;neurons\;x\;average\;synapse\\ number\;per\;neuron \end{array}$$ (2)

The average number of neurons in the human brain and the average number of synapses per neuron can be obtained from abundant literature studies whereas the storage required for a single synapse is determined from the synaptic morphology model considered below.

### 2.1 Average numbers of neurons and synapses per neuron

The exact numbers of neurons and synapses in the human brain are challenging to precisely determine due to the brain’s complexity and variability and a brief, nearly 150-year, history of cell counting in human brains along with types of counting methods are presented by von Barthel [15]. Because of large biological variability among individual human brains and different brain regions as well as diverse cell counting methods employed, the number of neurons in the human brain and its parts reported in various studies highly ranges. For instance, in an average brain, a normal biological variation in the number of neocortical neurons occurs by a factor more than two [15].

Many textbooks, original articles, and reviews have affirmed that the total number of neurons in the human brain is one hundred billion [16–24]. This affirmation is also present in the textbook by Kandel et al. [18] which is a commonly used textbook in neuroscience (and even praised as the “bible of neuroscience” [15]) authored by a Nobel Prize laureate in physiology or medicine 2000. This textbook knowledge has been challenged by more robust methods for neuron number counting providing an estimate of 86.1 billion neurons [25]. In some other sources, the number of neurons in the entire human brain was estimated at 30 billion [26], 70–80 billion [27], 85 billion [28], 67–86 billion [29], and 75–125 billion [30].

The number of neurons in the cerebral cortex reported in various studies ranges from 2.6 billion [31] to 32 billion [32]. A majority of studies estimate this number between 10–20 billion neurons including 14 billion [33],10–19 billion [34], 18.8 billion [35], 12.4 billion [25], and 12.7 billion [29].

In the cerebellar cortex Haug estimated about 50 billion neurons [27] and Williams and Herrup 65–70 billion neurons [28], whereas Andersen et al. estimated 102 billion neurons in the human cerebellum (105 billion cells including 3 billion glial cells) [36]. Several studies imply that the range of 50–70 billion cerebellar neurons is the most credible, including 54 billion [29] and 69 billion [25].

The number of neurons together in the brainstem, striatum, and diencephalon is approximately 700 million [25]. The above studies are summarized in Table 1.

**Table 1:** Number of neurons in the average adult human brain (in billions).

Region	Minimum number of neurons	Maximum number of neurons	Number of neurons for majorities of studies
**Entire brain**	30 [26]	138 (by adding max components)	70–80 [27] 85 [28] 67–86 [29] 86.1 [25] 100 [16–24] 75–125 [30]
**Cerebral cortex**	2.6 [31]	32 [32]	14 [33] 10–19 [34] 18.8 [35] 12.4 [25] 12.7 [29]
**Cerebellum**	50 [27]	102 [36]	54 [29] 69 [25] 65–70 [28]
**Brainstem/striatum/** **diencephalon/**	0.7 [25]		

For the storage estimation, we take 4 values approximating the number of neurons in the entire brain, minimum 30, maximum 138 (obtained by adding maximum values for the cortex, cerebellum, and brainstem/striatum/diencephalon), 86, and 100 billion.

Many studies revealed that synapses are complex and diverse [37]. Moreover, their number can vary between individuals and change in response to learning and experience. Therefore, any estimates of their number should also be taken as an approximation.

The number of synapses per neuron is also highly variable. Kandel et al. estimate that there are around 10^14^ (100 trillion) synapses in the average adult human brain [18] (meaning 1,000 synapses per neuron). Ref. [38] reports that the total number of synapses in the human neocortex is approximately 0.15×10^15^ (0.15 quadrillion). According to a reference book [39], the average neuron has 1,000 synapses with other neurons. Ten thousand connections per neuron are reported in [23]. Some sources estimate that a single neuron can have between 1,000–15,000 synaptic connections [24,40]. Defelipe demonstrated that the number of synapses per neuron is also cortical layer-dependent and the study of the synaptic profiles within cubes of cortical tissue of 50 μm wide by 50 μm thick revealed that for 158 neurons and 4,483,400 synapses examined in 6 cortical layers, the average number of synapses per neuron was 29,642 (100,042 in layer I (for 5 neurons only), 17,046 in layer II, 37,066 in layer IIIa, 56,521 in layer IIIb, 15,989 in layer IV, 29,965 in layer V, and 28,224 in layer VI) [41].

Hence, for the storage estimation, we take 3 values, 1,000, 10,000, and 30,000 synapses per neuron.

### 2.2 Synaptome morphology definition and modeling

To describe neuronal morphology at the nanoscale a dedicated nano neuronal format *nN* has been introduced [42] and accommodated here to model the synaptome. Let us assume that the whole brain has been imaged and all its neurons are already processed, and their synaptic axonal and dendritic terminals (also termed elements or membranes) are extracted and located in some Cartesian coordinate system. Let the terminal morphology be characterized by the coordinates of its center, radius, and identifier. Hence, the morphology of a dendritic terminal is described as follows

Dendritic terminal identifier (*D*)

Dendritic terminal coordinates (*x*,*y*,*z*)

Dendritic terminal radius (*r*)

The morphology of an axonal terminal is described in the following way

Axonal terminal identifier (*A*)

Axonal terminal coordinates (*x*,*y*,*z*)

Axonal terminal radius (*r*)

**Fig 1:**
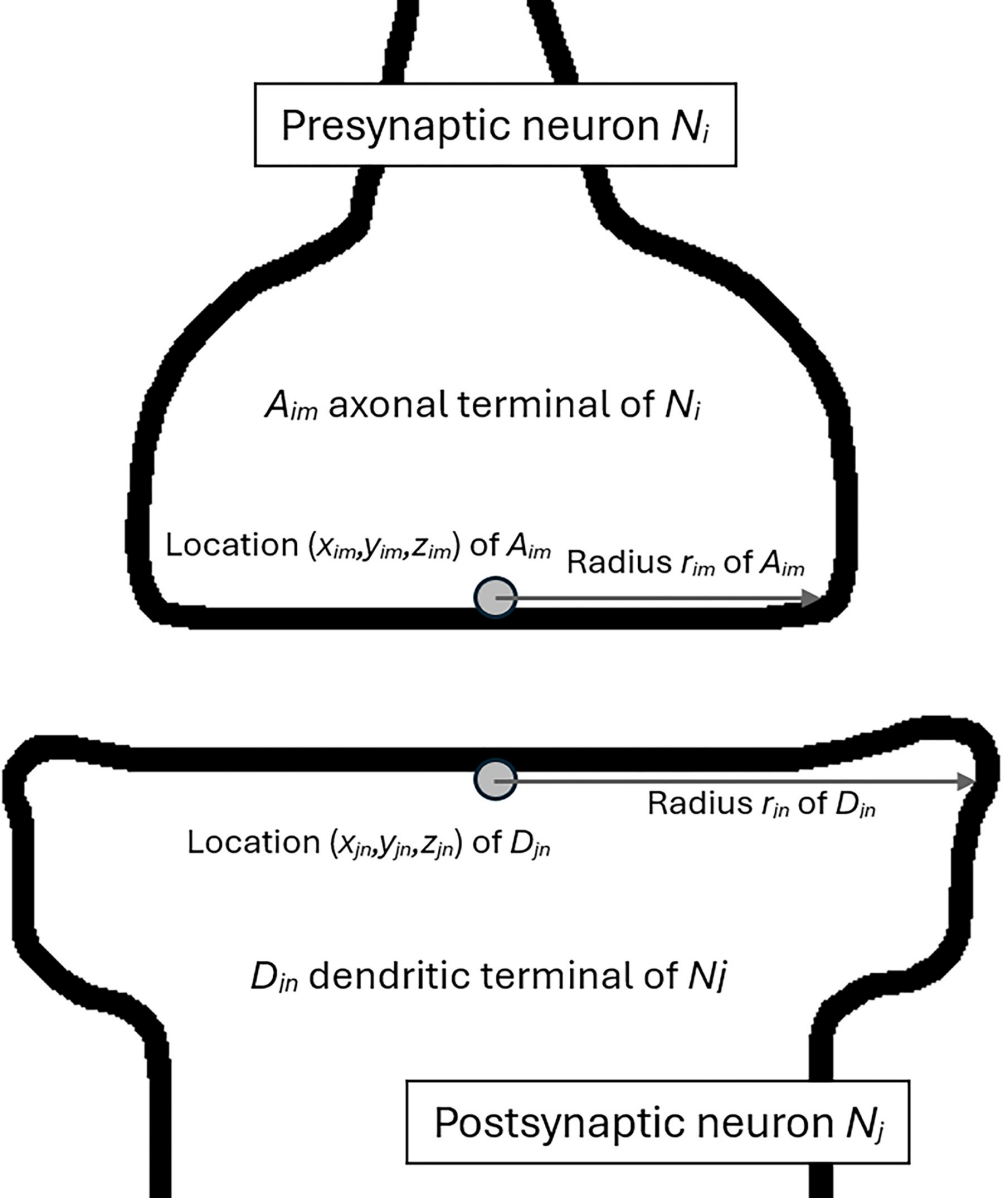
Synapse and its parameters.

Consider two neurons forming a synapse meaning the following pair of pairs [(presynaptic neuron),(presynaptic axonal terminal);(postsynaptic neuron),(postsynaptic dendritic terminal)]. Let *i* and *j* be a presynaptic neuron and the postsynaptic neuron with identifiers *N*_*i*_ and *N*_*j*_, and *m* and *n* the axonal terminal *A*_*im*_ and the dendritic terminal *D*_*jn*_ of neurons *i* and *j*, respectively. Then, the synapse between the neurons *i* and *j* is defined as a pair of quadruples [*N*_*i*_,*A*_*im*_,(*x*_*im*_,*y*_*im*_,*z*_*im*_),*r*_*im*_;*N*_*j*_,*D*_*jn*_,(*x*_*jn*_,*y*_*jn*_,*z*_*jn*_),*r*_*jn*_]. A synapse has three components, topology (the neuron and terminal identifiers), location (the coordinates of the terminal centers), and geometry (the terminal radii by approximating the fundi of the terminals with the circular shape), Fig 1. The complete synaptome is defined as a set of all the synapses {[*N*_*i*_,*A*_*im*_,(*x*_*im*_,*y*_*im*_,*z*_*im*_),*r*_*im*_;*N*_*j*_,*D*_*jn*_,(*x*_*jn*_,*y*_*jn*_,*z*_*jn*_),*r*_*jn*_]} for all the presynaptic and the corresponding postsynaptic neurons.

I consider three synaptic morphology models with diverse content and potential applications, namely, (1) the topologic model with topology, (2) the point model with topology and location, and (3) the geometric model with topology, location, and geometry.

The topologic model encapsulates the topology of all presynaptic neurons along with the topology of the corresponding postsynaptic neurons, i.e., {(presynaptic topology);(postsynaptic topology)}. The point model characterizes the topology and location of all presynaptic neurons and the corresponding postsynaptic neurons, i.e., {(presynaptic topology and location);(postsynaptic topology and location)}. The geometric model describes the topology, location, and geometry of all presynaptic neurons and the corresponding postsynaptic neurons, i.e., {(presynaptic topology, location, and geometry);(postsynaptic topology, location, and geometry)}.

### 2.3 Single synapse storage assessment

For the four considered cases of the number of neurons i.e. 30, 86, 100, and 138 billion, we get {3 10^10^, 86 10^10^, 10^11^, 1.38 10^11^} = {2^34.80^, 2^36.32^, 2^36.54^, 2^37.01^}. Hence, to store the neuron’s unique identifier out of other 30, 86, 100, or 138 billion neurons, 5 bytes (B) are needed in any of these four cases. For the three considered cases of the number of synapses per neuron, i.e., 1,000, 10,000, 30,000, we get {10^3^, 10^4^, 3 10^4^} = {2^9.97^, 2^13.29^, 2^14.87^}. Hence, for a given neuron to store its terminal’s unique identifier out of other 1,000, 10,000, or 30,000 terminals, 2 B are required in any of these three cases.

The terminal coordinates are voxel resolution and synapse type dependent. There are two types of synapses, chemical with the synaptic gap (cleft) of 20-30/20-40 nm [43,44] and electrical with the 2–4 nm gap [43]. To distinguish the synapses, the acquisition data shall be sampled sufficiently dense and according to the Nyquist sampling theorem, the spatial sampling resolution shall be no lower than 10 nm to handle chemical synapses and 1 nm for the electrical synapses. To distinguish values at the levels of 10 nm and 1 nm spatial resolutions, the required ranges of values shall be 10^8^ (2^26.6^) for the 10 nm resolution and 10^9^ (2^29.9^) for the 1 nm resolution. Hence, 4 B are needed to store a terminal single coordinate value for each of the 10 nm and 1 nm voxel resolutions. To store a terminal location with 3 coordinates,12 B of storage are required. To store a terminal radius, 1 number meaning 4 B are needed. Hence, a single synapse storage determined by the synaptic model as a pair of (neuron, terminal) pairs requires

$$\begin{array}{l} •\;14\;(2x5+2x2)\;B\;for\;the\;topologic\;model;\\ •\;38\;(2x5+2x2+2x3x4)\;B\;for\;the\;point\;model;\\ •\;46\;(2x5+2x2+2x3x4+2x4)\;B\;for\;the\;geometric\;model. \end{array}$$ (3)

These values, because of an enormous number of synapses, will result in huge storage requirements so three simplifications are proposed to the synaptic model. First, in the full synaptic model for a given presynaptic neuron with identifier *N*_*i*_ its all presynaptic terminals *T*_*im*_ (each including the identifier, coordinates, and radius) are represented as a sequence of {[*N*_*i*_,*T*_*i1*_] … [*N*_*i*_,*T*_*im*_] … [*N*_*i*_,*T*_*iM*_]}, where *iM* is the number of presynaptic terminals in neuron *N*_*i*_. Since the neuron identifier is the same for all its presynaptic terminals, this sequence can be expressed as (*N*_*i*_,{*T*_*im*_}), m = 1 … *iM*. The number of presynaptic terminals is estimated as follows. Every presynaptic terminal (the presynaptic neuron’s output) has its corresponding postsynaptic terminal (the postsynaptic neuron’s input) so, consequently, the number of all neuronal inputs equals the number of all neuronal outputs. Therefore, on average we can assume that in an average neuron, half of its terminals are presynaptic and the other half postsynaptic. As the storage required for the presynaptic neuron’s terminals is for the considered cases at least three orders higher than that for the presynaptic neuron’s identifier, so the latter can be neglected. Second, the two sets of coordinates for the corresponding presynaptic and postsynaptic terminals can be approximated by a single set of coordinates with their mean value. Similarly, the presynaptic and postsynaptic terminal radii can be approximated by their mean value. Hence, the single synapse storage requirements for the simplified synapse model are the following

$$\begin{array}{l} •\;9\;(1x5+2x2)\;B\;for\;the\;topologic\;model;\\ •\;21\;(1x5+2x2+1x3x4)\;B\;for\;the\;point\;model;\\ •\;25\;(1x5+2x2+1x3x4+1x4)\;B\;for\;the\;geometric\;model. \end{array}$$ (4)

## 3 Results

To estimate the storage required for the human synaptome Eq 2 is employed, where the single synapse storage for the full version of the synaptic model is determined by relationship (3) and for the simplified version of the synaptic model by relationship (4), the numbers of neurons in the average adult human brain and its parts are given in Table 1, and the numbers of synapses per neuron are provided in Section 2.3. To accommodate for a wide range of variations in the numbers of neurons and synapses reported in numerous sources, four cases of neurons and three cases of synapses per neuron are considered. Additionally, taking into account three synaptic models with full and simplified versions, in total 72 cases providing the storage required for the synaptome are estimated.

For 100 billion neurons each on average with 10 thousand synapses 0.5 x 14 x 10^11^ x 10^4^ = 7 x 10^15^ = 7 petabyte (PB) storage is required for the full topologic model. The full point and geometric models need 19 PB and 23 PB, respectively. The simplified models require 4.5 PB, 10.5 PB, and 12.5 PB for the topologic, point, and geometric models, respectively. For 86 billion neurons each on average with 10 thousand synapses these storage requirements reduce to 6.02 (3.87) PB, 16.34 (9.03) PB, and 19.78 (10.75) PB for the full (simplified) topologic, point, and geometric models, respectively. For 86 billion neurons each on average with one thousand synapses the storage requirements are 0.6 (0.39) PB, 1.63 (0.9.) PB, and 1.98 (1.08) PB for the full (simplified) topologic, point, and geometric models, respectively. The storage requirements for all 72 cases are given in Table 2.

**Table 2:** Storage in PB required for the human synaptome for the given number of neurons and synapses per neuron estimated for the full topologic, point, and geometric synaptic models (and the corresponding simplified models in parentheses).

Number of synapses per neuron	30 billion neurons	86 billion neurons	100 billion neurons	138 billion neurons
**1,000**	0.21 (0.14) topologic 0.57 (0.32) point 0.69 (0.38) geometric	0.60 (0.39) topologic 1.63 (0.90) point 1.98 (1.08) geometric	0.70 (0.45) topologic 1.90 (1.05) point 2.30 (1.25) geometric	0.97 (0.62) topologic 2.62 (1.45) point 3.17 (1.73) geometric
**10,000**	2.10 (1.35) topologic 5.70 (3.15) point 6.90 (3.75) geometric	6.02 (3.87) topologic 16.34 (9.03) point 19.78 (10.75) geometric	7.00 (4.50) topologic 19.00 (10.50) point 23.00 (12.50) geometric	9.66 (6.21) topologic 26.22 (14.49) point 31.74 (17.25) geometric
**30,0000**	6.30 (4.05) topologic 17.10 (9.45) point 20.70 (11.25) geometric	18.06 (11.61) topologic 49.02 (27.09) point 59.34 (32.25) geometric	21.00 (13.50) topologic 57.00 (31.50) point 69.00 (37.50) geometric	28.98 (18.63) topologic 78.66 (43.47) point 95.22 (51.75) geometric

The simplified version of the synaptic model provides 1.56 (14/9), 1.81 (38/21), and 1.84 (46/25) storage reduction of the corresponding full topologic, point, and geometric models, respectively.

Similarly, by applying relationships (2)-(4) and Table 1, storage requirements for different parts of the brain can be estimated. Then, Table 3 provides the storage required for the cerebral cortex, cerebellum, and brainstem/striatum/diencephalon for the case of 86 billion neurons in the entire brain (for consistency all the numbers are from the same source [25]) estimated for the full and simplified topologic, point, and geometric synaptic models.

**Table 3:** Storage required for the human cerebral cortex (in TB), cerebellum (in PB), and brainstem/striatum/diencephalon (in TB) for the case of 86 billion neurons in the entire brain for the given number of synapses per neuron estimated for the full topologic, point, and geometric synaptic models (and the corresponding simplified models in parentheses).

Number of synapses per neuron	Cerebral cortex (in TB)	Cerebellum (in PB)	Brainstem/striatum/ diencephalon (in TB)
**1,000**	86.80 (55.80) topologic 235.60 (130.02) point 285.20 (155.00) geometric	0.48 (0.31) topologic 1.31 (0.72) point 1.59 (0.86) geometric	4.90 (3.15) topologic 13.30 (7.35) point 16.10 (8.75) geometric
**10,000**	868.00 (558.00) topologic 2,356.00 (1,302.00) point 2,852.00 (1,550.00) geometric	4.83 (3.11) topologic 13.11 (7.25) point 15.87 (8.63) geometric	49.00 (31.50) topologic 133.00 (73.50) point 161.00 (87.50) geometric
**30,0000**	2,604.00 (1,674.00) topologic 7,068.00 (3,906.00) point 8,556.00 (4,650.00) geometric	14.49 (9.32) topologic 39.33 (21.74) point 47.61 (35.86) geometric	147.00 (94.50) topologic 399.00 (220.50) point 483.00 (262.50) geometric

The same approach can, in general, be employed to estimate the data required for small brains, however, Eqs (3)–(4) are not directly applicable because the size of the synapse identifier is neuron and synapse number dependent.

Let us estimate storage requirements for *C*.*elegans* [5]. To distinguish 7 x 10^3^ = 2^12.77^ synapses, 2B are required for the synapse identifier (with no need to apply the neuron identifier). Then, the single synapse storage needs 4 (2x2) B for the topologic model, 28 (2x2 + 2x3x4) B for the point model, and 36 (2x2 + 2x3x4+ 2x4) B for the geometric model. Hence, the full synaptic model of *C*.*elegans* with about 7,000 synapses requires around 28 KB for the topologic model, 196 KB for the point model, and 252 KB for the geometric model. For the simplified synaptic model, the single synapse storage needs 2 (1x2) B for the topologic model, 14 (1x2 + 1x3x4) B for the point model, and 18 (1x2 + 1x3x4+ 1x4) B for the geometric model. Therefore, the simplified synaptic model requires about 14 KB for the topologic model, 98 KB for the point model, and 126 KB for the geometric model.

Let us estimate storage requirements for *Drosophila*. Scheffer et al. report that *Drosophila* has about 100,000 neurons and that the central hemibrain consists of approximately 25,000 neurons and around 20 million chemical synapses [45]. To estimate the storage required for *Drosophila* I use the information about the data size of the *FlyWire* dataset which is an electron microscopy reconstruction of the complete brain of an adult female *Drosophila* with about 130 thousand neurons and 32 million synapses representing the complete *Drosophila* brain [46–49]. Therefore, to distinguish 32 x 10^6^ = 2^24.93^ synapses, 4B are required for the synapse identifier. Then, the single synapse storage needs 8 (2x4) B for the topologic model, 32 (2x4 + 2x3x4) B for the point model, and 40 (2x4 + 2x3x4+ 2x4) B for the geometric model. Hence, the full synaptic model of *Drosophila* with 32 million synapses requires 256 MB for the topologic model, 1,024 MB for the point model, and 1,280 MB for the geometric model. For the simplified synaptic model, the single synapse storage needs 4 (1x4) B for the topologic model, 16 (1x4 + 1x3x4) B for the point model, and 20 (1x4 + 1x3x4+ 1x4) B for the geometric model. Therefore, the simplified synaptic model requires 128 MB for the topologic model, 512 MB for the point model, and 640 MB for the geometric model.

The comparison of storage required for C.*elegans*, *Drosophila*, and human (for the case of 86 billion neurons in the entire brain and 1,000 synapses per neuron) estimated for the full and simplified topologic, point, and geometric synaptic models is presented in Table 4.

**Table 4:** Comparison of storage required for C.*elegans*, *Drosophila*, and human (for the case of 86 billion neurons in the entire brain and 1,000–10,000 synapses per neuron) estimated for the full topologic, point, and geometric synaptic models (and the corresponding simplified models in parentheses).

	*C*.*elegans* (kB)	*Drosophila* (in MB)	Human (in PB)
**Storage**	28 (14) topologic 196 (96) point 252 (126) geometric	256 (128) topologic 1,024 (512) point 1,280 (640) geometric	0.60–6.02 (0.39–3.87) topologic 1.63–16.34 (0.90–9.03) point 1.98–19.78 (1.08–10.75) geometric

To evaluate the proposed method, the first large, nanoscale human brain tissue sample of a 1 mm^3^ [50] is employed here since a volumetric image dataset of the entire human brain at the nanoscale has not yet been acquired. The sample was surgically acquired from the temporal lobe of the cerebral cortex, stained, embedded in resin, cut into ~30 nm thick sections, and scanned using electron microscopy. The resulting volume comprises 50,000 cells, hundreds of millions of neurites, and 150 million synapses. In [51] this volume was additionally annotated such that each neuron object had 19 attributes and each synapse object had seven attributes (synapse ID, synapse type, presynaptic site considered as an object, postsynaptic partner considered as an object, location, bounding box, and layer). To store the annotated properties of the synapses along with those of the neurons and layers, nearly 27 GB of storage was required.

In our synaptic model, a synapse is characterized by identifier, location, and size whereas in [51] a synapse is annotated with seven attributes, so to enable a fairer comparison the geometric model is extended with 14 additional numbers, 12 for the four coordinates of a rectangular bounding box and one for each of synapse type and layer. Hence, the extended geometric model requires 46 + 14 x 4 = 102 B. This storage still may be underestimation, as here I consider the presynaptic and postsynaptic sites to be represented by indexes whereas in [51] they are represented as objects.

The extended geometric model for 150 million synapses requires 102 x 150 x 10^6^ = 15,300 x 10^6^ = 15.3 GB. Taking into account that this extended geometric model is likely underestimation and that nearly 27 GB cited in [51] is needed not only for the synapses but also for the annotated cells and hundreds of millions of neurites, these two amounts of storage are roughly comparable.

## 4 Discussion

One of the fundamental challenges in neuroscience is to generate the human nanoscale connectome. The key groups of challenges are data acquisition and computing. The acquisition produces big data and requires an enormous amount of time as already mentioned in the introduction.

For very small brains, such as for the *Drosophila* brain, the acquired dataset can cover the entire adult brain, while in mammals the largest datasets include only a tiny fraction of a brain. For instance, a complete adult *Drosophila* brain was imaged with electron microscopy resulting in 7,050 40 nm thin sections forming a dataset of about 106 TB [46]. Regarding mammals, a tissue sample of the mouse lateral geniculate body having 67 million cubic microns of 400 x 600 x 280 resolution with about 100 trillion voxels yielded a dataset of about 100 TB [52]. A sample of 1 mm^3^ of mouse neocortex with 26,500 electron microscopy ultrathin sections with 4 nm/pixel resolution yielded a dataset of more than 2 petabytes [53]. Hence, a sample of 500 mm^3^ would have required a 1 exabyte storage. This dataset was continuously imaged across five microscopes for almost 6 months, each producing a daily throughput of 3.6 TB [53]. Not only acquisition but also validation is time consuming. To validate a hemibrain center of *Drosophila* assuming a rate of 1000 connections annotated per trained person, per day, would have taken more than 230 working years [45]. The first large nanoscale human brain tissue sample with 5,292 slices of 33 nm average thickness and 4 x4 nm^2^ pixels yielded a volume of approximately 2.1 PB [50].

The key computational challenges to achieve this goal are big data [2] and high-performance computing [3]. Although suitable data enabling the computation of the entire human connectome at the nanoscale have not been acquired yet, some of the computational challenges have already been addressed earlier [42,54,55]. I have previously formulated a dedicated nano neuronal data file format to describe neuron morphology at the nanoscale [42], estimated storage requirements to keep the volumetric and four geometric neuronal models (straight wireframe, enhanced wireframe, straight polygonal, and enhanced polygonal) [54], estimated high performance computing requirements for morphology modeling of the entire human brain at the nanoscale [55], and designed a nanoscale human brain atlas enabling the exploration of the brain model at the nanoscale [42].

As the connectome can be computed from the synaptome, toward the synaptome calculation I here present three morphologic models of the human nanoscale synaptome and quantitatively estimate the data bigness for storing them. These synaptome models are topologic, point, and geometric. Having the synaptome determined, any neuronal microcircuit is formed as a sequence of synapses whereas all the microcircuits, in turn, constitute the connectome. Any synaptic model introduced here enables the calculation of the connectome.

The numbers of neurons and synapses in the human brain and its parts are highly variable and widely range in numerous studies reported; therefore, here I consider four cases of the total number of neurons in the entire brain including the minimum and maximum, and three cases of synapses per neuron in addition to the full and simplified versions of three synaptic models, topologic, point and geometric resulting in overall 72 cases. The storage required for the entire brain is estimated in the range from 30 billion neurons and 1 thousand synapses per neuron to 138 billion neurons and 30 thousand synapses per neuron. Then the full synaptic model of the entire human brain requires from 0.21 PB to 28.98 PB for the topologic model, from 0.57 PB to 78.66 PB for the point model, and from 0.69 PB to 95.22 PB for the geometric model, Table 2. For the simplified synaptic model, the topologic model requires from 0.14 PB to 18.63 PB, the point model from 0.32 PB to 43.47 PB, and the geometric model from 0.38 PB to 51.75 PB, Table 2. The simplified model reduces the required storage to 64%, 55%, and 54% for the topologic, point, and geometric models, respectively.

Note that in all these cases the maximum storage required is 138 times higher than the minimum storage. This results from the fact that the minimum and maximum numbers of neurons are likely to be outliers and a more credible number is 86 billion neurons [25]. Similarly, the approximate number of 30 thousand synapses per neuron reported by Defelipe [41] was measured for a very small tissue sample, so the range of 1–10 thousand synapses per neuron is likely to be more credible. Then, the storage required is from 0.60 (0.39) PB to 6.02 (3.87) PB, from 1.63 (0.90) PB to 16.34 (9.03) PB, and from 1.98 (1.08) PB to 19.78 (10.75) PB for the full (simplified) topologic, point, and geometric models, respectively. Note that then the difference between the minimum and maximum storage is 10 times only.

The reported number of neurons in different parts of the brain also varies in the literature from 2.6 to 32 billion (12.31 times) for the cerebral cortex and from 50 to 102 billion for the cerebellum (2.04 times), Table 1. Taking the numbers of neurons from the same source [25], the cerebral cortex contains 14%, the cerebellum 80%, and the brainstem/striatum/diencephalon 0.8% of the total number of neurons in the entire human brain. Then, the full synaptic model of the cerebral cortex requires from 86.80 TB to 2.60 PB for the topologic model, from 235.60 TB to 7.07 PB for the point model, and from 285.20 TB to 8.56 PB for the geometric model, Table 3. For the simplified synaptic model of the cerebral cortex, the topologic model needs from 55.80 TB to 1.67 PB, the point model from 130.02 TB to 3.91 PB, and the geometric model from 155.00 TB to 4.65 PB, Table 3.

The full synaptic model of the cerebellum requires from 0.48 PB to 14.49 PB for the topologic model, from 1.31 PB to 39.33 PB for the point model, and from 1.59 PB to 47.61 PB for the geometric model, Table 3. For the simplified synaptic model of the cerebellum, the topologic model needs from 0.31 PB to 9.32 PB, the point model from 0.72 PB to 21.74 PB, and the geometric model from 0.86 PB to 35.86 PB, Table 3.

The full synaptic model of the brainstem/striatum/diencephalon requires from 4.90 TB to 147.00 TB for the topologic model, from 13.30 TB to 399.00 TB for the point model, and from 16.10 TB to 483.00 TB for the geometric model, Table 3. For the simplified synaptic model of the brainstem/striatum/diencephalon, the topologic model needs from 3.15 TB to 94.50 TB, the point model from 7.35 TB to 220.50 TB, and the geometric model from 8.75 TB to 262.50 TB, Table 3.

Note that the above synaptome storage estimations are valid for both the chemical and electrical synaptomes, as the single synapse storage covers the data sampling spatial resolutions of 10 nm and 1 nm.

A volumetric image dataset of the entire human brain at the nanoscale enabling the computation of the synaptome and connectome has not yet been acquired. Consequently, any validation of the considered synaptic model with the actual human data is not yet feasible. What is available today is the first large, nanoscale human surgical tissue sample of a 1 mm^3^ [50] which is employed here to assess the proposed method. Then, our geometric model extended to partly compensate for the seven synaptic annotation attributes in [51] requires 15.3 GB to store this sample whereas [51] reports nearly 27 GB to store the synapses together with the annotated cells and hundreds of millions of neurites. Therefore, these two amounts of storage can be considered roughly comparable. Note that the synaptic representation in [51] requires to store layers as acquired in electron microscopy, however, there is no need to store layers in synchrotron tomography data.

Moreover, this is not so straightforward to compare our estimated synaptome data bigness with the actually acquired datasets. The actually acquired datasets can include the raw images, segmentation data, annotations, reconstructions, and various representations like skeletons and connectivity graphs. In addition, the size of data can vary depending on the image resolution, extent of the imaged brain, data format, data simplification, and applied data compression. Note that even for *Drosophila*, synaptome completeness is beyond the current reach resulting in providing synapse counts that are representatives [45]. On the other hand, our model and storage estimates assume the completeness of the human synaptome.

Regarding small animal brains, the localization part of the synapse representation in *Drosophila* proposed by Scheffer et al. [45] corresponds to our synaptic point model. Namely, the synapses there are stored as point annotations meaning one point for a presynaptic T-bar (corresponding on electron microscopy images to an active zone of the presynaptic terminal), and one point for each of its postsynaptic densities in the postsynaptic terminal (note that some more complex synaptic structures found, e.g., in the cerebellum may exhibit multiple active zones and the corresponding multiple postsynaptic densities). However, the identifiers of the neurons containing their connecting synapses are determined from segmentation which assigns 64-bit neuron identifier to each voxel, meaning requiring 8 B for neuron and 6 B for voxel identifier to distinguish 26 teravoxels. Our point synaptic model scaled to the *Drosophila* size (see the Results section) requires only 4 B for the synapse identifier meaning it is 10 B smaller per synapse than the synaptic data representation employed by Scheffer et al. [45].

The same method applied here to estimate the storage required for the human synaptome can, in principle, also be used to estimate synaptomes of other animals after the compensation for the synapse identifier dependency on the numbers of neuron and synapses. Namely, the synaptoms of *Drosophila* and C.*elegans* are “merely” at the level of megabytes and kilobytes, respectively (Table 4) in comparison to the human synaptome which requires storage at the level of petabytes.

Even though the synaptic topologic model for the entire brain is much smaller than each of the synaptic point and geometric models, it is still too big to be stored on today’s top supercomputers related to neuroscience such as *Sequoia* of 1.5 PB and *K* of 1.4 PB of memory [2]. For the number of neurons in the range of 86–100 billion, only the case of 1,000 synapses per neuron can fit to this storage (and up to about 2,000 synapses per neuron) whereas the case of 10,000 synapses per neuron does not fit. Note that the simplified point and geometric models for the 1,000 synapses per neuron also fit this amount of storage. For the cerebral cortex with 12.4 billion neurons (Table 1) and in the range of 1,000–10,000 synapses per neuron, the full topologic and simplified point models fit the amount of memory provided by *Sequoia* or *K*. Today’s most powerful and world’s first exascale supercomputer *Frontier* [56] with 9,408 computing nodes each with 4 TB of flash memory offers an overall 37 PB memory. *Frontier*, for the synaptic model of the entire brain with 86 billion neurons and the 1,000–10,000 range of the number of synapses per neuron, is able to handle the human nanoscale synaptome, and for 30,000 synapses per neuron only the simplified topologic, point, and geometric models. Another exascale supercomputer *Aurora* with the aggregate system memory of 10.9 PB [57] for 86 billion neurons is able to handle only the simplified synaptic models up to 10,000 synapses per neurons.

The storage of the above supercomputers serves as a benchmark for our results. In general, the storage limits of the today’s supercomputers can be overcome either by a network of powerful supercomputers enabling to scale computing resources, such as *BRAINS* [58], or cloud storage offered by open-source services, such as the *Brain Observatory Storage Service and Database* which is a cloud-based solution for storing and accessing petascale image datasets [59].

The topologic model is sufficient and the most efficient to compute the connectome or rather its topology. The synaptic point model allows for the construction of the connectomic point model with the locations of the synapses. The synaptic geometric model enables the construction of the connectomic geometric model with the locations and sizes of the synapses. Note that these connectomic models do not allow to construct the exact full path between the consecutive synapses in a microcircuit as in the synaptic model the parameters of the soma, axon, and dendritic and axonal trees (as formulated in [42]) are not available (meaning can be discarded after the calculation of the synaptome).

The introduced synaptic models are useful for morphometric and network-related analyses. The topologic model allows for the determination of the number of microcircuits, loops, and feedbacks.

The point model enables the calculation of synaptic densities and patterns of interconnectivity such as distributions of microcircuits with respect to length, underlying structures and tracts, and local and global networks (subsystems). In particular, this includes the distribution of microcircuits among the cerebrum, cerebellum, brainstem, and subcortical structures as well as their distribution among the cerebral and cerebellar cortical layers. Other network-related features also can be calculated including clusters (hubs), worldness, betweenness centrality, and motifs.

The geometric model additionally enables the calculation of the distribution of the terminal sizes (provided that they, especially the postsynaptic terminals, are feasible to be determined).

Storage-wise this work is limited to the size of the already calculated synaptome. However, its calculation requires a much bigger storage to handle all the neurons along with their complete dendritic and axonal trees as estimated in [54]. In addition, synchrotron tomography requires also space for numerous projections from which the volumetric raw data are calculated. Depending on the spatial resolution, the volumetric raw data require from 2,800 exabytes (EB) for 10 nm resolution (to image chemical synapses) to 2,800,000 (EB) for 1 nm resolution (to image electrical synapses) [54]. By converting the volumetric raw data into a geometric model, the storage required, depending on the type of geometric model, ranges from 20.56 PB to 82.56 PB for 86 billion neurons and 10,000 synapses per neuron [54].

Model-wise this work is restricted to synaptome morphology although the synaptome size can be expected to be much higher at the molecular level. For instance, in [60] the mouse synaptome was considered at the molecular level by cataloging and mapping enormous synapse diversity. Namely, the postsynaptic proteome contains more than 1,000 proteins whereas as few as 10 proteins could produce 1,023 types and 10^11^ subtypes of excitatory synapses.

Neurodegenerative processes cause death of neurons and synapse loss, degeneration of synapses, and the spread of pathology through the brain via synapses. Therefore, the study of synaptome is also vital from the standpoint of synaptopathy, meaning the disturbances in neuronal connectivity, which with the increasing awareness is one of the first pathological even in many neurologic disorders [40]. Studies of synapses in Alzheimer’s disease (AD) [61–64], dementia with Lewy bodies [65], and amyotrophic lateral sclerosis [66] demonstrated changes in synaptic density and morphology as well as molecular composition and the aggregation of disease-associated proteins in the synapse which may disrupt its structure and function. In AD, the loss of synapses also correlates with a cognitive decline [61,63].

Moreover, some evidence indicates that synaptopathy underlines a variety of psychiatric disorders [67]. The involvement of synaptic disturbances in the pathophysiology of schizophrenia was reviewed by Obi-Nagata et al. [24]. The schizophrenic patients were observed to have the reduced dendritic spine density [68–70]. Glanz et al. [68] reported that in schizophrenic subjects, spine density in the prefrontal cortex was decreased by 23% compared with normal control subjects. Garey et al. [69] demonstrated the 66% dendritic spine density decrease in frontal regions and 59% decrease in temporal regions in cortical layer III. Konopaske et al. [70] observed that the number of spines per dendrite was reduced by 21.6% for the dorsolateral prefrontal cortex. The decrease in spine number was only observed for smaller spines and not for larger spines [71] resulting in less plastic circuits (as large spines are generally highly stable and small spines are more plastic and related to learning and behavioral flexibility [72]).

Therefore based on these studies, this is likely to expect the storage required for the synaptome of persons with neurologic disorders to be smaller than that needed for the normal subjects. It remains still unclear how generalizable are these findings across all cortical layers and the entire cerebral cortex. Nevertheless, assuming in average a 25% spine density decrease for the whole cerebral cortex and taking 86 billion neurons in the entire brain, the required storage for the diseased cerebral cortical synaptome would range from the minimum of 41.85 TB for the topologic simplified model with 1,000 synapses per neuron to the maximum of 6417 TB for the full geometric model with 30,000 synapses per neuron (compare with Table 3).

Finally comparing the human genome and synaptome, the storage required for the human genome varies (depending on coverage) from 30 GB to 200 GB [73] and it seems today rather “small” in comparison to that required for the human nanoscale synaptome that is at the level of petabytes. However, to store hundreds of thousands of human genomes it is estimated that 2–40 EB of storage capacity will be needed by 2025 [73]. As a similar trend may be expected in neuroscience, this estimation gives some idea of how enormous storage will be required to store hundreds of thousands of human synaptomes and connectomes.

## 5 Summary

A key challenge in neuroscience is to generate the human nanoscale connectome which requires knowledge of synaptome forming the neural microcircuits. The synaptic architecture determines the limits of individual mental capacity and provides the framework for understanding neurologic disorders. Toward this objective, I here address morphology modeling and storage estimation for the human synaptome at the nanoscale. The storage required for the human synaptome depends on the number of synapses and the storage necessary for a single synapse determined by a synaptic model. I here introduce three synaptic morphology models with diverse content and potential applications: topologic with topology, point with topology and location, and geometric with topology, location, and geometry. They are useful for morphometric and network-related analyses. The topologic model allows for the determination of the number of microcircuits, loops, and feedbacks. The point model enables the calculation of synaptic densities and patterns of interconnectivity. The geometric model additionally enables the calculation of the distribution of terminal sizes.

The number of neurons in the human brain and its parts reported in various studies highly ranges because of large biological variability among individual brains and different cerebral regions as well as diverse cell counting methods employed. To accommodate for a wide range of variations in the number of neurons and synapses reported in numerous sources, four cases of the number of neurons in the brain (30; 86; 100; and 138 billion) and three cases of synapses per neuron (1,000; 10,000; and 30,000) are considered with three synaptic models with the full and simplified (to reduce storage) versions resulting in total 72 cases for which the storage required for the synaptome is estimated.

The full(simplified) synaptic model of the entire human brain requires from 0.21(0.14) PB to 28.98(18.63) PB for the topologic model, from 0.57(0.32) PB to 78.66(43.47) PB for the point model, and from 0.69(0.38) PB to 95.22(51.75) PB for the geometric model. The full(simplified) synaptic model of the cerebral cortex needs from 86.80(55.80) TB to 2.60(1.67) PB for the topologic model, from 235.60(130.02) TB to 7.07(3.91) PB for the point model, and from 285.20(155.00) TB to 8.56(4.65) PB for the geometric model. The full synaptic model of the cerebellum requires from 0.48(0.31) PB to 14.49(9.32) PB for the topologic model, from 1.31(0.72) PB to 39.33(21.74) PB for the point model, and from 1.59(0.86) PB to 47.61(35.86) PB for the geometric model. The full (simplified) synaptic model of the brainstem/striatum/diencephalon requires from 4.90(3.15) TB to 147.00(94.50) TB for the topologic model, from 13.30(7.35) TB to 399.00(220.50) TB for the point model, and from 16.10(8.75) TB to 483.00(262.50) TB for the geometric model.

The topologic model is sufficient and the most efficient to compute the connectome or rather its topology, however, even though it is much smaller than each of the synaptome point and geometric models, it is still too big to be stored on today’s top supercomputers related to neuroscience such as *Sequoia* of 1.5 PB and *K* of 1.4 PB of memory (and for the number of neurons in the range of 86–100 billion, only neurons with up to about 2,000 synapses can fit to this storage). The most powerful world’s supercomputer *Frontier* for the entire brain with 86 billion neurons and the 1,000–10,000 number of synapses per neuron is able to handle the human nanoscale synaptome, and for 30,000 synapses per neuron only the simplified topologic, point, and geometric models. The storage limits of today’s supercomputers can be overcome either by a network of powerful supercomputers enabling to scale computing resources or cloud storage offered by open-source services.

The human synaptome requires storage at the level of petabytes whereas the synaptoms for *Drosophila* and C.*elegans* are “merely” at the level of megabytes and kilobytes, respectively.

In the future, to store hundreds of thousands of human synaptomes and connectomes, storage at the level of exabytes or higher will be required. To my best knowledge, this is the first big data effort attempting to provide storage requirement estimation for the human nanoscale synaptome.
